# Laparoscopic approach to intrapelvic nerve entrapments

**DOI:** 10.1093/jhps/hnv030

**Published:** 2015-06-06

**Authors:** Nucelio Lemos, Marc Possover

**Affiliations:** 1. Pelvic Neurodysfunction Clinic of the Department of Gynecology of the Federal University of São Paulo, São Paulo, Brazil; 2. Neuropelveology Department of the Klinik Hirslanden, Zurich, Switzerland

## Abstract

It is long known that a large portion of the lumbosacral plexus is located intra-abdominally, in the retroperitoneal space. However, most of literature descriptions of lesions on this plexus refer to its extra-abdominal parts whereas its intra-abdominal portions are often neglected. The objective of this review article is to describe the laparoscopic anatomy of intrapelvic nerve bundles, as well as the findings and advances already achieved by Neuropelveology practitioners.

## INTRODUCTION

It is long known that a large portion of the lumbosacral plexus is located intra-abdominally, in the retroperitoneal space [1]. However, most of literature descriptions of lesions on this plexus refer to its extra-abdominal parts whereas its intra-abdominal portions are often neglected [2].

In 2007, Possover *et al*. [3] described the Laparoscopic Neuronavigation (LANN) technique, opening the doors to accessing the retroperitoneal portion of the lumbosacral plexus through a safe, minimally invasive and objective way. Since then, multiple causes of intrapelvic nerve entrapments have been described and a new field in Medicine—the Neuropelveology—was created.

The objective of this review article is to describe the laparoscopic anatomy of intrapelvic nerve bundles, as well as the findings and advances already achieved by Neuropelveology practitioners.

## LAPAROSCOPIC ANATOMY OF THE INTRAPELVIC NERVES

### Ilio-hypogastric, ilio-inguinalis and genito-femoralis nerves

**Fig. 1.**
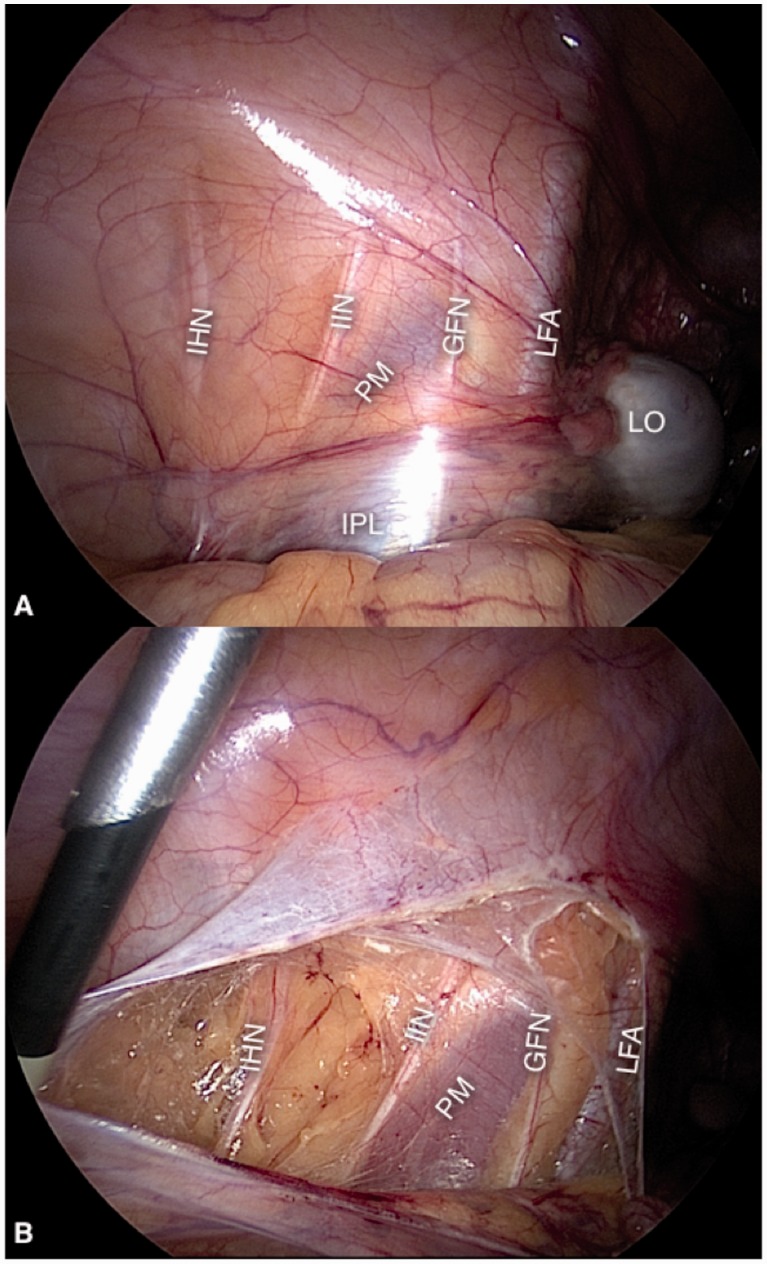
Ilio-hypogastric (IHN), ilio-inguinalis (IIN) and genito-femoralis (GFN) nerves, with the overlying peritoneum intact (A) and exposed (B). PM, psoas muscle; LO, left ovary; IPL, infundibulopelvic ligament; LFA, left femoral artery.

These nerves are sensitive branches of the lumbar plexus and enter the retroperitoneal space emerging on the lateral border of the psoas muscle and follow anteriorly and distally to leave the abdomen through the femoral and inguinal canals. Their fibrotic entrapment is related to post-herniorrhaphy inguinodynia [4] (Fig. 1).

### Femoral nerve

**Fig. 2.**
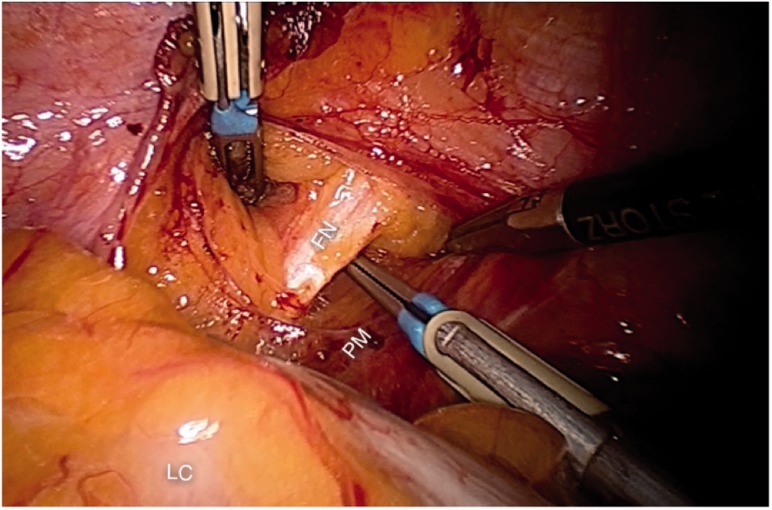
The left femoral nerve (FN) entering the retroperitoneal space on the posterolateral aspect of the psoas muscle (PM). LC, left colon.

The femoral nerve is the largest motoric and sensitive nerve of the lumbar plexus. It enters the abdomen by the postero-lateral aspect of the psoas muscle and leaves it through the femoral canal (Fig. 2) to innervate the quadriceps muscle and the skin covering the anterior thigh.

### Nerves of the obturator space

**Fig. 3.**
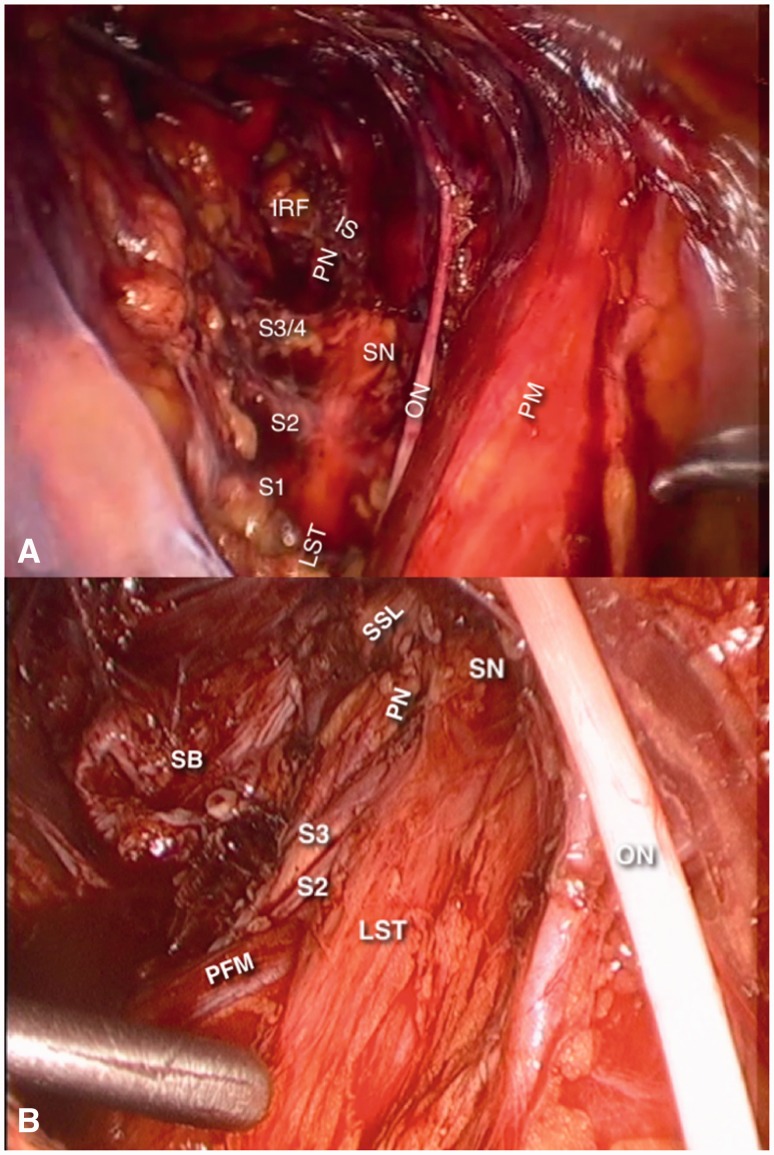
Nerves of the obturator space (right side). Picture (A) is the final aspect of a laparoscopic approach to Alcock’s Canal Syndrome, where the sacrospinous ligament has been transected to expose the pudendal nerve (PN). In picture B, the sacrospinous ligament (SSL) is intact. In both pictures, the internal and external iliac vessels are retracted medially. ON, obturator nerve; PM, psoas muscle; SN, sciatic nerve; LST, lumbosacral trunk; PN, pudendal nerve; IRF, ischiorectal fossa; IS, ischial spine; SB, sacral bone; PFM, pyriformis muscle.

The obturator nerve enters the obturator space at the level of the pelvic rim and leaves it through the obturator canal. It gives sensitive branches to the skin of the medial thigh and motoric branches to the hip adductors (Fig. 3A).

The lumbosacral trunk and the distal portions of the S1, S2, S3 and S4 nerve roots merge into the obturator space and form the sciatic and pudendal nerves (Fig. 3B).

The sciatic nerve is formed by the L4 and L5 fibers of the lumbosacral trunk and fibers from the S1, S2 and S3 nerve roots and leaves the pelvis through the sciatic notch. It gives out sensitive branches to the upper gluteal region, postero-lateral thigh, leg ankle and foot. It also controls the hip extensors, abductors and rotators, knee flexors and all the muscles for the ankle and foot.

The pudendal nerve is formed by fibers of the second, third and fourth nerve roots and leaves the pelvis through the pudendal (Alcock’s) canal. It gives out sensitive branches to the lower gluteal region and the perineal skin. It also sends motoric branches to the perineal muscles and the anterior fibers of the levator ani muscles.

Finally, there are direct motoric and sensitive nerves from the S3 and S4 nerve roots to the posterior fibers of the levator ani muscle.

### Nerves of the presacral and pararectal spaces

**Fig. 4.**
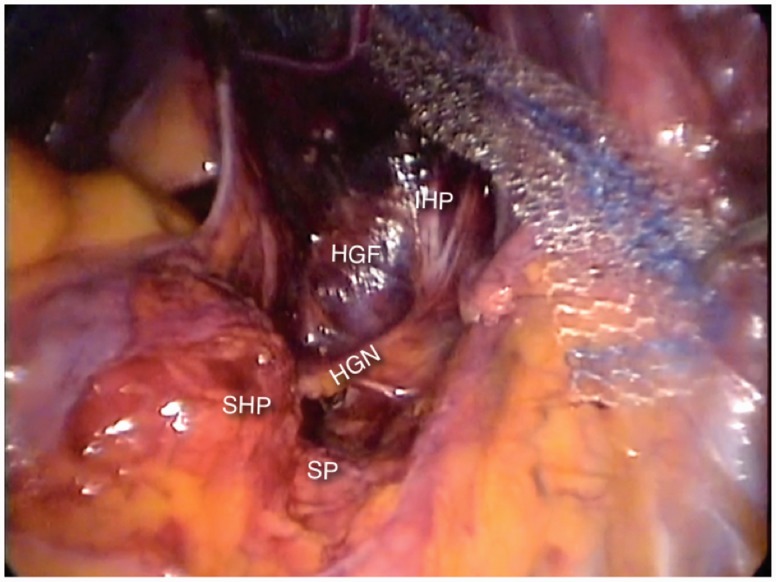
The hypogastric nerve (HN) emerges from the superior hypogastric plexus (SHP) at the level of the sacral promontory (SP) and runs anteriorly and distally, juxta-laterally to the hypogastric fascia (HF), to merge with the pelvic splanchnic nerves to form the inferior hypogastric plexus (IHP).

The superior hypogastric plexus, which is formed by fibers from para-aortic sympathetic trunk and gives rise to the hypogastric nerves. The hypogastric nerves run over the hypogastric fascia in an anterior and distal direction. After crossing about two thirds of the distance between the sacrum and the uterine cervix or the prostate, its fibers spread to join the pelvic splanchnic nerves (described later) to form the inferior hypogastric plexus (Fig. 4). The hypogastric nerves carry the sympathetic signals to the internal urethral and anal sphincters, rectum and bladder, which cause detrusor relaxation and bladder contraction, thus promoting continence. They also carry proprioceptive and nociceptive afferent signals from the pelvic viscerae.

**Fig. 5.**
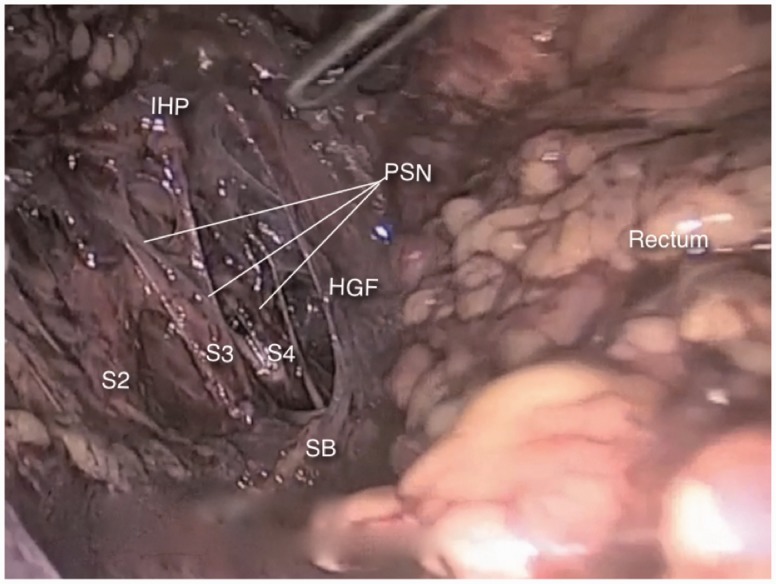
The sacral nerve roots (S2–S4) can be found juxta-laterally to the hypogastric fascia (HGF) and give origin to the pelvic splanchnic nerves (PSN), which run anteriorly and distally to merge the hypogastric nerve and form the inferior hypogastric plexus (IHP).

The lateral limit of the presacral space is the hypogastric fascia, which is the formed by the medial most fibers of the endopelvic fascia. The sacral nerve roots can be found juxta-laterally to this fascia (Fig. 5). They leave the sacral foramina and run anteriorly and distally, lying over the pyriformis muscle and crossing the internal iliac vessels laterally to them, to merge and form the nerves of the sacral plexus. Before crossing the internal iliac vessels, they give out the thin parasympathetic branches called pelvic splanchnic nerves, which promote detrusor contraction and provide extrinsic parasympathetic innervation to the colon descendens, sigmoid and rectum. They also carry nociceptive afferent signals from the pelvic viscerae. The pelvic splanchnic nerves join the hypogastric nerves to form the inferior hypogastric plexus in the pararectal fossae.

## INTRAPELVIC NERVE ENTRAPMENT SYNDROME

### Definition and symptoms

Nerve entrapment syndrome, or compression neuropathy, is a clinical condition caused by compression on a single nerve or nerve root. Its symptoms include pain, tingling, numbness and muscle weakness on the affected nerve’s dermatome [5]. Intrapelvic nerve entrapments are, therefore, entrapments of the intrapelvic portions of the nerves described in the previous sessions and will produce symptoms related to their dermatomes.

The above definition refers to the entrapment of somatic nerves. Autonomic nerve entrapment will produce visceral and vegetative symptoms, such as urinary frequency or urgency, dysuria, rectal pain, suprapubic and/or abdominal cramps and chills.

However, as described earlier, the sacral nerve roots give origin to both somatic and parasympathetic nerves. Therefore, entrapments of these roots will produce pain on their somatic dermatomes, together with urinary and bowel dysfunction.

In a concise manner, the main symptoms of intrapelvic nerve entrapments are:

– Sciatica associated with urinary symptoms (urgency, frequency, dysuria) or without any clear orthopedic cause;
– Gluteal pain associated with perineal, vaginal or penile pain;
– Dysuria and/or painful ejaculation;
– Refractory urinary symptoms;
– Refractory pelvic and perineal pain.

It is important to emphasize that, due to the distance between both plexuses, intrapelvic nerve entrapments will usually cause unilateral symptoms.

## ETIOLOGY OF INTRAPELVIC ENTRAPMENTS

### Endometriosis

The first report of intrapelvic nerve entrapment was made by Denton and Sherill [6], who described a case of cyclic sciatica due to endometriosis in 1955. After that, some other case reports and small series were published, until 2011, when Possover *et al*. [2] described the largest series so far, with 175 patients, all treated laparoscopically.

In endometriotic entrapments, the symptoms tend to be cyclic, worsening on the premenstrual and menstrual days and ameliorating or even disappearing on the post-menstrual period [2, 7, 8].

**Fig. 6.**
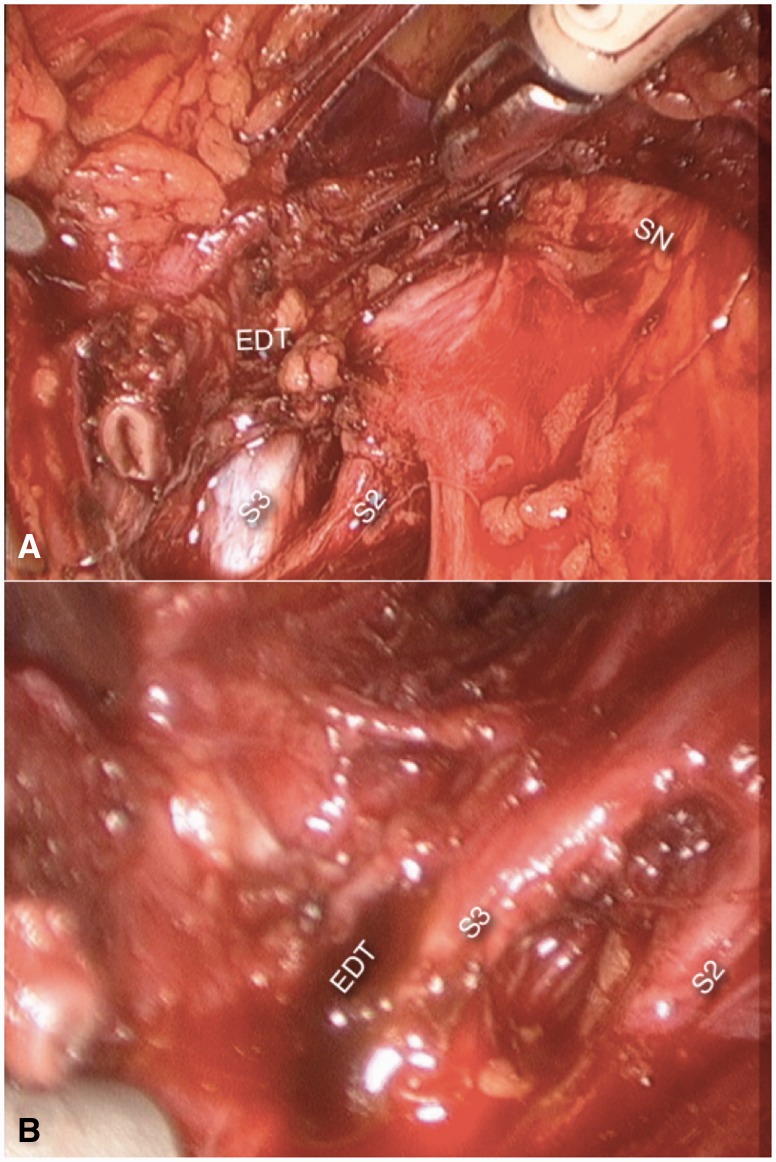
Endometriosis (EDT) involving nerve Roots S2 and S3 and the sciatic nerve (SN). Further dissection (B) revealed an endometriotic cyst (EDT) in S3.

Treatment consists of preoperatively identifying the symptoms and determining the topographical localization of the lesions (by means, mainly, of anamnesis and neurological examination and, sometimes, by MRI) and laparoscopically exploring all suspect segments of the plexus, with radical removal of all endometriotic foci and fibrosis [2, 7, 8] (Fig. 6).

The true incidence of endometriosis involving the sacral plexus is unknown, as this presentation of the disease is often neglected. In average, patients undergo four surgical procedures seeking to treat the pain before getting the right diagnosis [2]. Moreover, ∼40% of women with endometriosis refer unilateral pain on the inferior limb [9] and, in 30% of patients with endometriosis, leg pain was demonstrated to be neuropathic [10], which leads to the conclusion that endometriotic involvement of the lumbosacral plexus is probably underdiagnosed and much more frequent than reported.

### Fibrosis

This is one of the most frequent causes of intrapelvic nerve entrapments and possibly the most well-known etiology, since Amarenco [11] described the pudendal neuralgia in cyclists, in whom the pain is a consequence of fibrotic entrapment due to continued trauma.

**Fig. 7.**
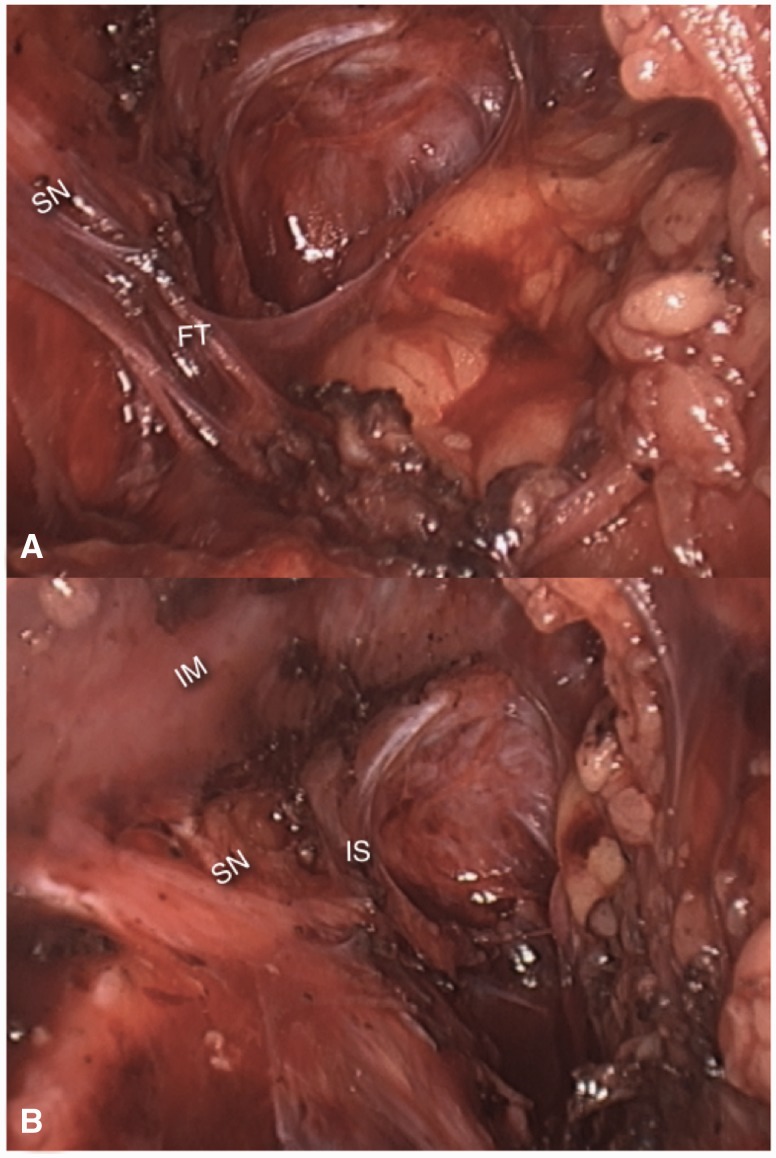
(A) Dense fibrotic tissue (FT) entrapping the left sciatic nerve (SN) against the pyriformis muscle. (B) final aspect after neurolysis. IM, iliac muscle; IS, ischial spine).

Despite the historical aspect, however, surgical manipulation seems to be the most frequent cause of fibrosis over the sacral plexus (Fig. 7). Among the surgeries with higher risks of inducing such kinds of entrapments are the pelvic reconstructive procedures [12].

### Vascular entrapment

Pelvic congestion syndrome is a well-known cause of cyclic pelvic pain. Patients commonly present with pelvic pain without evidence of inflammatory disease. The pain is worse during the premenstrual period and pregnancy, and is exacerbated by fatigue and standing [13].

**Fig. 8.**
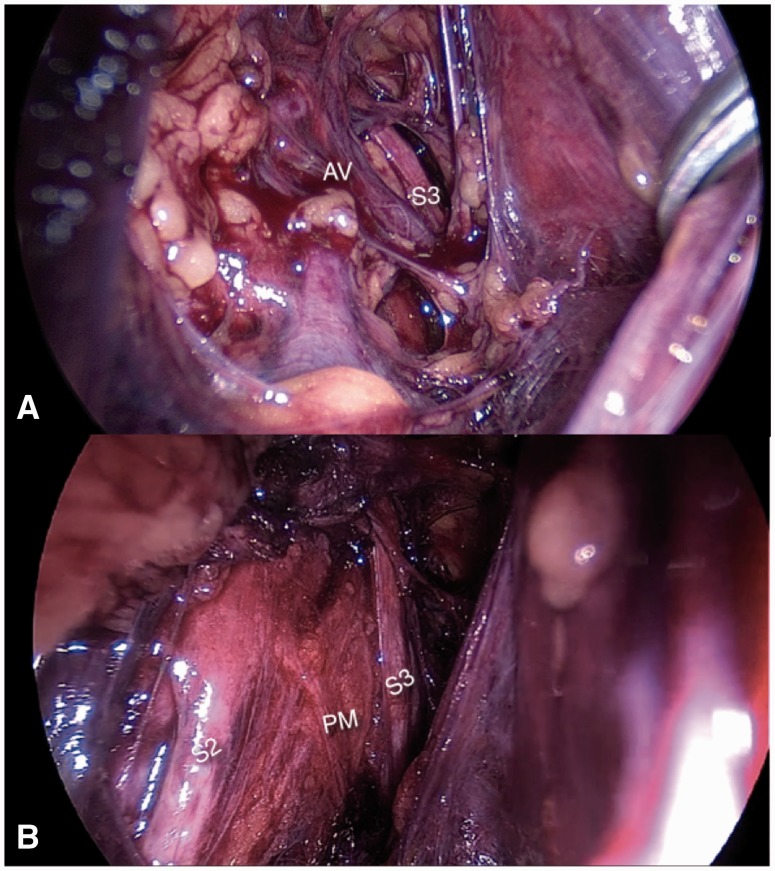
(A) Abnormal varicose vein (AV) entrapping S2 and S3 nerve roots against the pyriformis muscle (PM).

However, what is much less known is the fact that dilated or malformed branches of the internal or external iliac vessels can entrap the nerves of the sacral plexus against the pelvic sidewalls, producing symptoms such as sciatica, or refractory urinary and anorectal dysfunction [2, 14] (Fig. 8).

### Pyriformis syndrome

**Fig. 9.**
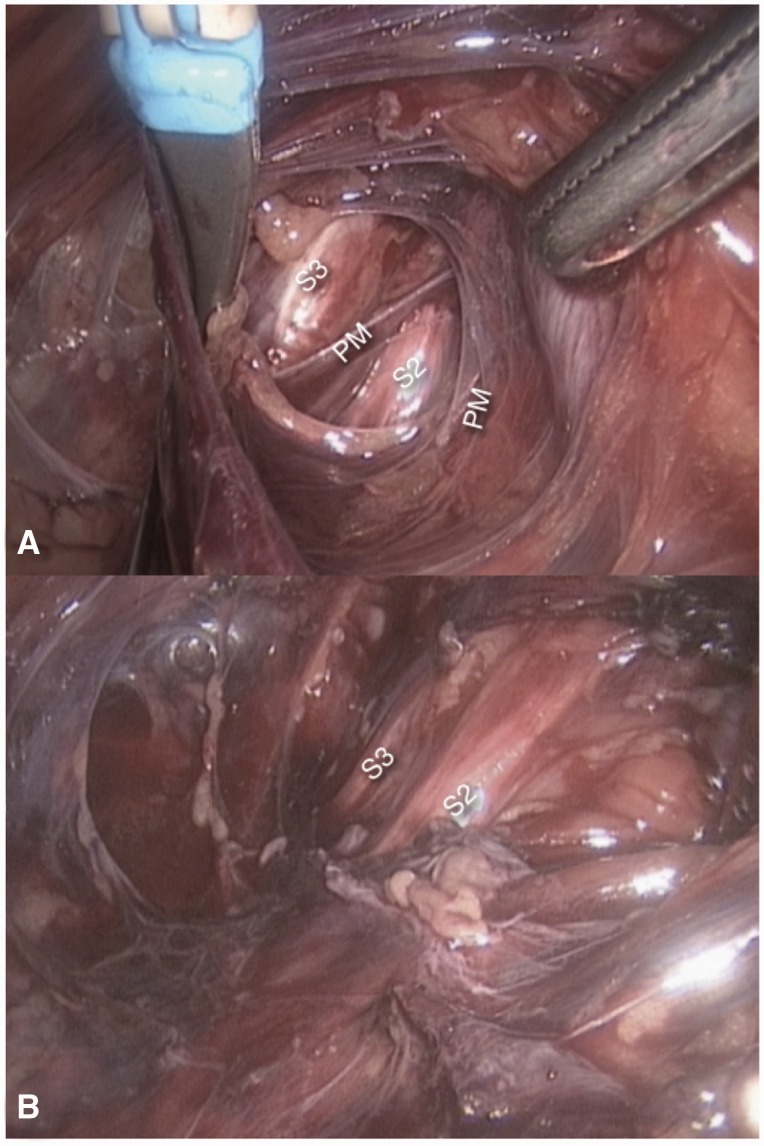
S2 and S3 nerve roots entrapped in between the pyriformis muscle (PM) fibers.

Numerous malformations of the pyriformis muscle have been described in the deep gluteal space that can entrap branches of the sciatic nerve. The laparoscopic approach has revealed that the intrapelvic fibers of this muscle can also entrap the sacral nerve roots [15]. Usually, these fibers originate from the sacral bone, laterally to the sacral foramina; some people present with some of the pyriformis fibers originating medially to the sacral foramina and involving the sacral nerve roots (Fig. 9). Differentiating intrapelvic from extrapelvic pyriformis syndrome can be very challenging. Bowel and urinary symptoms are a good indication that the entrapment is intrapelvic, but these are not always present.

### Primary neuropathic pain, nerve transection and secondary neuropathic pain

All the previously described causes of intrapelvic neuropathies have extrinsic entrapment as the etiology of pain. Intrapelvic radiculopathies can also result from intrinsic dysfunctions of the nerves themselves.

Nerve transections can occur during surgery or trauma and can induce neuroma formation, resulting in phantom pain and anesthesia of the affected nerve dermatome. The pictorial example of this is the phantom pain secondary to amputations, where branches of the sciatic and femoral nerves are transected. In the same fashion, pudendal transection will induce perineal pain and perineal anaesthesia, as well as unilateral atrophy of perineal muscles, usually resulting in urinary and fecal incontinence.

In entrapment syndromes chronic ischemia induces cytoarchitectural changes to the neuron, which do not heal properly after detrapment, resulting in neuropathic pain. The latter the detrapment is performed, the higher the risk of neuropathic pain.

Neuropathic pain can also result from metabolic disturbances of the neuron, infectious agents, chronic exposure to neurotoxic substances or a myriad of other causes.

Finally, in many cases, although the topography of the lesion is determined, its etiology cannot be identified.

In all of these situations, the laparoscopic implantation of neuromodulation can be used to specifically modulate the affected nerve, producing very encouraging results when compared with the more commonly available epidural neuromodulation [4,15].

**Fig. 10.**
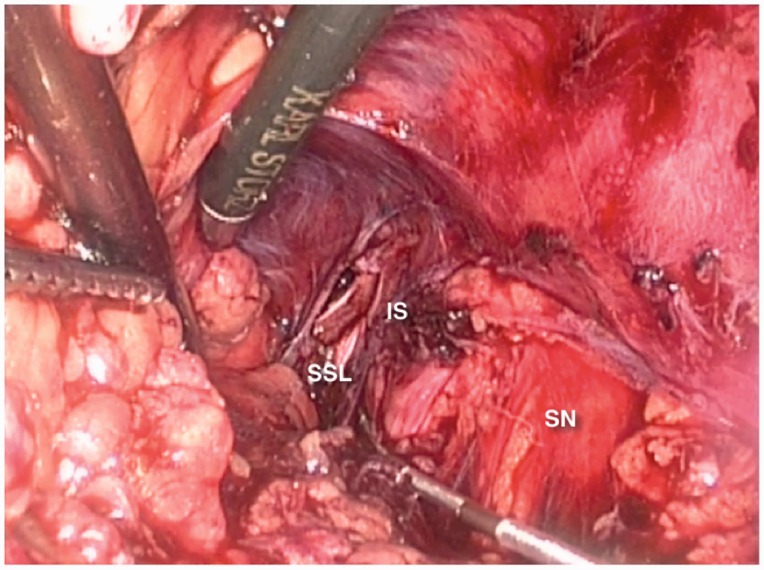
LION electrode placed on right sciatic and pudendal nerves. PM, psoas muscle; IS, ischial spine; SN, sciatic nerve; SSL, sacrospinous ligament.

The laparoscopic implantation of neuroprosthesis—the LION procedure—was first reported by Possover in 2009 as a rescue procedure in patients with local complications of a Brindley procedure [16]. Due to its successful results and lesser invasiveness, it was then used as a primary procedure in spinal cord-injured patients, aiming to improve locomotion and bladder function [17]. Long-term data has shown improvement in voluntary motor function and sensitivity, suggesting positive effects on neuroplasticity [18] (Fig. 10).

## CONCLUSION

The laparoscopic approach to the intrapelvic bundles of the lumbosacral nerves opened a myriad of possibilities to assess and treat this neglected portion of this nerve plexus, by means of nerve decompression or selective neuromodulation.

When facing sciatica, gluteal or perineal pain without any obvious spinal or deep gluteal causes, the examiner should always remember that the entrapment could be in the intrapelvic portions, manly when urinary symptoms are present.

## CONFLICT OF INTEREST STATEMENT

Nucelio Lemos received research grants from Medtronic Inc. and Laborie Inc. and travel and proctorship grants from Medtronic Inc. None of these grants are, however, directly related to the current publication.
